# The ameliorating effect of exercise on long-term memory impairment and dendritic retraction via the mild activation of AMP-activated protein kinase in chronically stressed hippocampal CA1 neurons

**DOI:** 10.20463/jenb.2018.0022

**Published:** 2018-09-30

**Authors:** Yea-Hyun Leem, Hyukki Chang

**Affiliations:** 1 Department of Molecular Medicine and Tissue Injury Defense Research Center, Ewha Womans University, Seoul Republic of Korea; 2 Department of Human Movement Science, Seoul Women's University, Seoul Republic of Korea

**Keywords:** Chronic stress, regular exercise, AMPK, hippocampal CA1, neuronal remodeling, memory consolidation

## Abstract

**[Purpose]:**

Chronic stress affects the neuronal architecture of hippocampal subfields including the Cornu Ammonis 1 (CA1) region, which governs long-term memory. Exercise exerts a beneficial effect on memory improvement via hippocampal AMP-activated protein kinase (AMPK) activation. However, the relationship between the two phenomena is poorly understood. This study used animal and cell culture experimental systems to investigate whether chronic stress-induced impairment of memory consolidation and maladaptation of the neuronal architecture in the hippocampal CA1 area is prevented by regular exercise through AMPK activation.

**[Methods]:**

Mice underwent four weeks of treadmill running with or without a 6h/21d-restraint stress regimen, along with treatment with Compound C. Memory consolidation was assessed using the Morris Water Maze (MWM). Dendritic rearrangement of hippocampal CA1 neurons was evaluated using the Golgi-Cox stain and Sholl analysis. Additionally, the primary hippocampal culture system was adopted for in vitro experiments.

**[Results]:**

Chronic stress-induced failure of memory retention and reduction in AMPK activation were ameliorated by the exercise regimen. Chronic stress-or repeated corticosterone (CORT)-provoked malformation of the neuronal architecture was also suppressed by both exercise and treatment with 5-aminoimidazole-4-carboxamide ribonucleotide (AICAR).

**[Conclusion]:**

Chronic stress causes dendritic retraction among dorsal hippocampal CA1 neurons via the downregulation of AMPK activation, thereby leading to failure of memory retention. In contrast, regular exercise protects against chronic stress-evoked defects in memory consolidation and changes in neuronal morphology in the dorsal hippocampal CA1 area via mild activation of AMPK.

## INTRODUCTION

Chronic stress is a risk factor for cognitive-and/or mood-related behavioral abnormalities that are involved in abnormal synaptic plasticity in the dorsal hippocampus^1,2^.

A large body of evidence has demonstrated that chronic stress alters structural and functional plasticity in some limbic structures such as the prefrontal cortex (PFC), hippocampus, and amygdala^3-6^. Chronic stress-induced morphological and molecular alterations in PFC neurons are similar to those reported in the hippocampus^7-9^, suggesting that chronic stress results in aberrant structural plasticity such as dendritic shrinkage in the PFC and hippocampus, which leads to impaired cognitive-related behaviors.

Chronic stress or repeated treatment with stress hormones such as corticosterone caused aberrant structural plasticity in pyramidal neurons, which was evidenced by decreased dendritic growth, ramification, and spine density in hippocampal subareas^10,11^. The dorsal hippocampal Cornu Ammonis 1 (CA1) region is a key structure that regulates memory consolidation^1^. Some studies suggest that chronic stress-induced decline in synaptic efficacy among hippocampal CA1 neurons may be responsible for the observed abnormal structural plasticity. This leads to long-term memory consolidation failure^11-13^. One conceivable mechanism underlying hippocampus-dependent memory persistence is the structural plasticity of dendrites, which is a key component in determining synaptic properties such as synaptic efficacy and excitatory neurotransmission.

AMPK is widely expressed in the central nervous system, including the hippocampus, and is activated upon phosphorylation in response to increase in local intracellular Ca^2^+ levels, ATP depletion, metabolic stress, and exercise^14-16^. The positive role of hippocampal AMPK activation in cognitive and mood-related abnormalities has been demonstrated^17-20^. Recently, we demonstrated that hippocampal AMPK activation after treatment with 5-aminoimidazole-4-carboxamide ribonucleotide (AICAR), an AMPK activator, improved learning and memory as well as sustained memory^16^. The aforementioned findings suggest that hippocampal AMPK activity plays a regulatory role in stress-related behaviors which involve synaptic plasticity such as memory formation and persistence.

We recently reported that chronic stress-elicited impairment in memory function was restored/prevented by regular exercise via hippocampal AMPK activation in chronically-stressed mice^16^. However, a mechanistic and quantitative understanding of the role of exercise-induced AMPK activation in the dendritic morphology of dorsal hippocampal CA1 neurons during chronic stress has not been elucidated. Accordingly, we investigated whether the dendritic morphology of hippocampal CA1 neurons is regulated by AMPK activity in chronic stress conditions using in vivo and in vitro experiments.

## METHODS

### Experimental mice

Seven-week-old male C57BL/6 mice were obtained from Daehan Biolink, Co. Ltd. (Eumsung, Chungbuk, Korea) and housed in clear plastic cages under specific pathogen-free conditions and a 12:12-h light-dark cycle (lights on at 08:00 and off at 20:00). The mice had ad libitum access to standard irradiated chow (Purina Mills, Seoul, Korea). The Animal Care and Use Committee of the Seoul Women’s University approved all experimental procedures involving animals.

### Experimental design

**Fig. 1.**
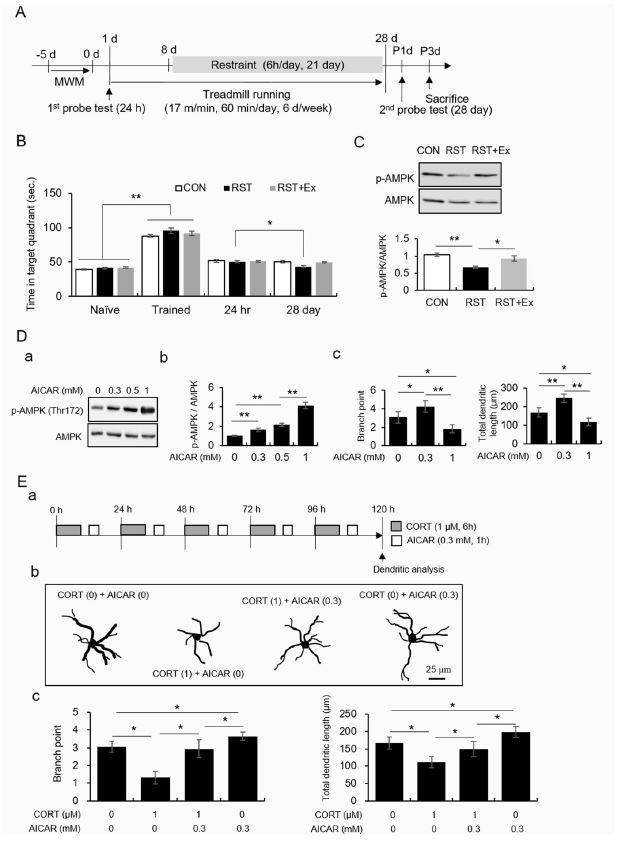
Regular exercise prevents chronic stress-induced failure of memory consolidation and changes in AMPK activity in the hippocampal CA1 area. A low-dose, and not a high dose, of AICAR enhanced dendritic branching and length in primary hippocampal cells treated with CORT. A. Experimental procedure. B. The quantitative analysis of memory consolidation measured using the Morris water maze. C. The quantitative analysis of AMPK and pAMPK protein levels. Photomicrograph showing western blot (upper panel). Quantification of pAMPK/AMPK (lower panel). D. The quantitative analysis of AMPK, pAMPK, dendritic branches, and dendritic length. (a) western blot data, (b) quantification of pAMPK/AMPK and quantification of dendritic branch points (c) and length (d). E. The quantitative analysis of dendritic branches and dendritic length. (a) experimental design, (b) Golgi-Cox stained and reconstructed neurons, (c) quantification of dendritic branch points and length. Data are presented as the means □ SEM. * and ** denote differences at p < 0.05 and p < 0.01, respectively.

In experiment 1 (Figure 1A-B), mice were divided into three groups: the control (CON), restraint stress (RST), and exercise combined with restraint stress (RST+Ex) groups. Mice were acclimated to the new surroundings for one week before the start of the experiment. The exercise protocol (which involved running a distance of 17 m/min, 60 min/day, 6 days/week for 4 weeks) was designed according to our previous study. Mice were subjected to restraint stress a week before the beginning of treadmill running. Mice were subjected to chronic restraint stress as described previously^16^. Mice performed the Morris Water Maze (MWM) task. Mice (n = 12/group) underwent a training session and two probe trials for memory consolidation. In experiment 2 (Figure 1D-E), to verify the regulatory role of AMPK activity on the morphology of hippocampal neurons following repeated exposure to the stress hormone, primary hippocampal cells were treated with AICAR (Tocris Bioscience, Bristol, UK) for an hour followed by the six-hour treatment with CORT (Sigma-Aldrich, MO, USA) at 1 μM in two-hour intervals for 120 hours to determine neuronal morphology.

**Fig. 2.**
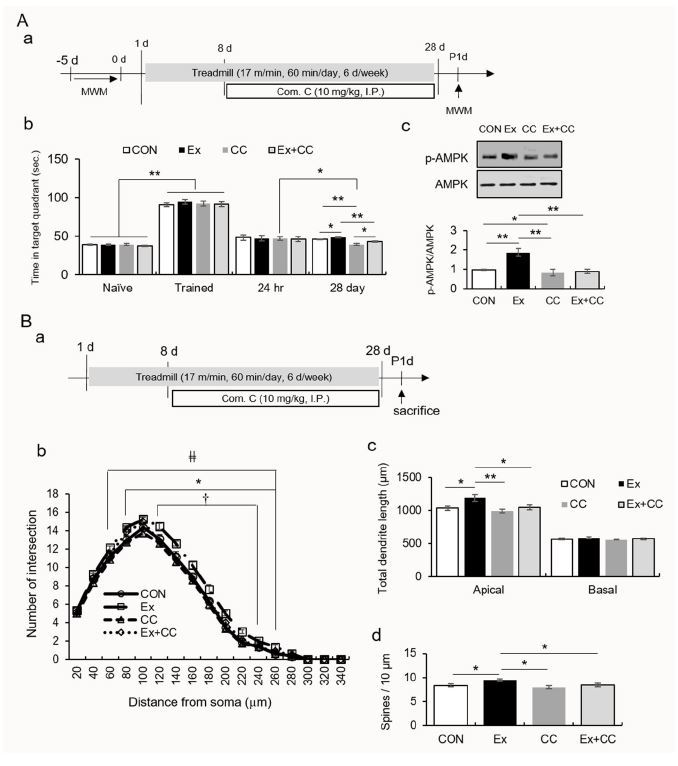
Regular exercise-induced AMPK activation resulted in long-term memory consolidation and enlarged dendritic structure. A. The quantitative analysis of memory consolidation measured by the Morris water maze. (a) Experimental design, (b) quantification of memory consolidation, (c) quantification of pAMPK/AMPK. B. The quantitative analysis of dendritic branches, dendritic length, and spine density. (a) Experimental design and quantification of dendritic intersection (b), total dendritic length (c), and spine number (d).

In experiment 2 (Figure 2), to elucidate the mechanism underlying the potential role of regular exercise-induced AMPK activation on morphological changes in hippocampal CA1 neurons, mice were divided into four groups: the control (CON), exercise (Ex), compound C treatment (CC), and exercise combined with compound C treatment (Ex-+CC) groups. Mice were intraperitoneally injected with compound C, a potent and selective inhibitor of AMPK, (10 mg/kg; EMD Chemicals, Gibbstown, NJ, USA) one hour before being subjected to treadmill running every two days during the exercise regimen. Mice without compound C treatment were injected with saline (1% dimethyl sulfoxide). Mice performed the MWM task as described in experiment 1 (n = 12/group). For Golgi staining, mice were sacrificed one day after the last exposure to stress in an independent experiment (n = 8/group). To evaluate the proteins, tissues from the stratum lacunosum moleculare (SLM) of the dorsal hippocampus, which corresponded to the region between -1.34 mm and -2.46 mm from the bregma (n =8/group), was used.

### Behavioral tests

The mice were randomly placed in the different quadrant (NW, NE, SE, or SW) where the platform was always located in a 1.5-m wide black circular pool containing water at a temperature of 22 °C. One training block was conducted on days 1 and 6. Two training blocks separated by a two-hour interval were conducted on days 2–5. Each training block comprised four training trials, with a two-minute inter-trial interval during which each mouse was placed at a random starting location in the pool, and was allowed to swim to the platform within a period of 120 s. If a mouse fails to reach the platform in the first 30 s, it was guided to the platform and allowed to remain on the platform for 30 s. The probe tests, which were conducted 24 h after the last training block and 28 days after the previous probe trial, required mice to swim for 60 s from a random starting location in a pool without a platform. The time spent in the target quadrant and the escape latency were monitored using the SMART 3.0 program (Panlab, S.L.U., Barcelona, Spain) on a computer, which was connected to a ceiling-mounted camera directly above the pool.

### Golgi staining, neuronal reconstruction, and morphometric analyses

Golgi-Cox staining of brain tissue was performed using a NovaUltraTM Golgi-Cox Stain Kit (IHC World, Woodstock, MD, USA) according to the procedure suggested by the manufacturer. The protocols for Golgi staining, reconstruction, and morphometric analyses have been described in a previous study published by our group^28^. On average, 6–8 neurons from each brain were selected based on the degree of preservation and visibility of the soma and dendritic trees.

### Synaptosome extraction and western blot analyses

Tissues obtained from mice were pooled (two-three mice) and homogenized. The procedures for synaptosome fractionation and western blot analysis have been described in a previous study published by our group^23^. The membranes were washed in the buffer and incubated with a horseradish peroxidase-conjugated secondary antibody (anti-rabbit IgG, 1:3000) for 2 h at 26 ℃. The optical density of each band was measured using Image J. Anti-AMPK (1:2000) and anti-phospho-AMPK (1:1000) were obtained from Cell Signaling Technology, Inc. (Danvers, MA, USA).

### Primary hippocampal culture

Primary hippocampal cell cultures from E17 (embryo 17 day) ICR mice were prepared. The procedure for hippocampal cell culture has been described in a previous study published by our group^20^.

### Statistical analysis

Statistical analysis was performed using the SPSS statistical software (SPSS for Windows, version 18.0; IBM Corporation, Armonk, NY, USA). One-way analysis of variance, two-way repeated measures analysis of variance, and independent t-tests were performed to assess significance. Post hoc comparisons were performed using Newman-Keuls tests. All values are reported as mean± standard error of the mean (SEM). Statistical significance was set at p < 0.05.

## RESULTS

### Regular treadmill running prevented chronic stress-induced memory consolidation impairments and downregulated AMPK activity in the dorsal hippocampal CA1

The performances (time in the target quadrant) of the naïve mice were enhanced by training (Figure 1B; t_70_ = -24.73, p < 0.01). Twenty-eight days after the first probe test, only the performance of the RST mice was significantly decreased compared to their performance 24 h after the last training trial (t_22_ = 2.64, p < 0.05), while the performances of the CON and RST+Ex groups did not significantly differ between these two trials (Figure 1B; CON: t_22_ = -0.43, p > 0.05; RST+Ex: t_22_ = 1.13, p > 0.05). The AMPK activity in the dorsal hippocampal CA1 of the RST mice was significantly decreased, and this decrease was similar to that in controls after treadmill running (Figure 2Ca and e; F_2, 6_ = 36.65, p < 0.01).

### Repeated CORT treatments resulted in dendritic rearrangement and these deficits were reversed by treatment with AICAR, a potent AMPK activator, in a rimary hippocampal culture

AICAR enhanced AMPK activity in a dose-dependent manner (Fig. 2Ab; F_3, 12_ = 151.80, p < 0.01). AICAR (0.1 mM) enhanced the dendritic length and branch points, while a high dose of AICAR (1 mM) caused dendritic retraction in primary hippocampal cells (Figure 2Ac; branch points: F_2_, 9 = 18.14, p < 0.01; dendritic length: F_2_, 9 =33.42, p < 0.01). The branch points and total dendritic lengths were significantly reduced by CORT treatment, and these decreases were reversed by treatment with AICAR (Figure 2Bc; branch points: F_3, 12_ = 30.23, p < 0.01; dendritic length: F_3, 12_ = 16.25, p < 0.01). Treatment with AICAR alone enhanced dendritic branching and length compared to those of control neurons (Figure 2Bc).

### Regular treadmill running altered the AMPK-mediated dendritic reorganization of CA1 neurons

In the MWM task, the performance of mice treated with compound C alone significantly decreased (t_22_ = 2.77, p < 0.05), while the performances of the CON, Ex, and Ex+CC groups did not (Figure 3A). On day 28, the amount of time that the mice which underwent treadmill running spent in the quadrant increased relative to those of the other groups (F_3, 44_ = 2.77, p < 0.01). The performance of the CC group was attenuated compared to those of the other groups (F_3, 44_ = 14.60, p < 0.01). The synaptosomal levels of p-AMPK in the SLM were significantly increased by treadmill running, and these increases were reversed by compound C (Figure 3Ac; F_3, 8_ = 23.96, p < 0.01). Treadmill running resulted in an increased dendritic arborization in a wide range of segments (80–260 μm), dendritic outgrowth, and spine density in the apical layer. AMPK inactivation prevented these increases (Figure 3B; dendritic intersection group × distance, F_48, 448_ = 1.43, p < 0.05; group, F_1, 28_ = 18,446.41, p < 0.01; distance, F_16, 448_ = 1.436.40, p < 0.01; apical dendritic length, F_3, 28_ = 5.73, p < 0.01; basal dendritic length, F_3, 28_ = 0.74, p > 0.05; spine number, F_3, 28_ = 3.84 p < 0.05).

## DISCUSSION

The current study demonstrated that chronic stress caused memory consolidation failure and reduced AMPK activity in the hippocampal CA1 region, which was prevented by regular exercise. Neuronal dendrites retracted due to repeated treatment with CORT. This shrinkage was restored by treatment with AICAR, an in vitro AMPK activator.

We found that regular exercise rescues chronic stress-induced failure of long-term memory and downregulation of AMPK activity. The CA1 area is a crucial subfield which governs hippocampal-dependent memory retention^1^. AMPK has been extensively studied as a core candidate molecule as AMPK has an exercise mimic effects. AMPK is an evolutionarily conserved regulator which senses metabolic changes, including local intracellular Ca^2^+ levels, ATP depletion, metabolic stress, and exercise^14,15^. Accumulating evidence has demonstrated that hippocampal AMPK activation improved cognitive functions^17,18^. Furthermore, several studies showed that chronic stress attenuates hippocampal AMPK activity and that the anti-depressive action of ketamine requires hippocampal AMPK activation in a chronic stress-based depression model^10,19,20,28^. The aforementioned information supports our hypothesis that AMPK activity in the hippocampal CA1 area may contribute to the beneficial effects of regular exercise against chronic stress.

A large body of evidence demonstrated chronic stress-induced neuronal remodeling in limbic structures such as the hippocampus^3-6,26,27^. Specifically, the diminished synaptic efficacy of hippocampal CA1 neurons was suggested to be related to abnormal structural plasticity which results in failure to consolidate long-term memory^12,13^. The postsynaptic architecture of excitatory synapses is regarded as an important component of connectivity between neurons, ultimately modulating neuronal excitability^13,28^. Based on the information provided by the aforementioned studies, our result on the restoration of AMPK activity in the hippocampal CA1 area by regular exercise in chronically stressed mice led us to assess the effects of AMPK activity on dendritic geometry in primary hippocampal culture. Interestingly, we found that a low-dose of AICAR enhanced dendritic branching and dendritic length, while a high-dose of AICAR had the opposite effect. In addition, repeated treatment with CORT caused the retraction of dendrites of hippocampal neurons. This was reversed by the mild activation of AMPK (Figure 2B). These results suggest that the persistent increase in CORT levels causes atrophy of hippocampal neurons and this change may be prevented by the mild activation, and not a higher activation, of AMPK.

To buttress available in vitro data, we explored the effects of regular exercise on the dendritic architecture of dorsal hippocampal CA1 neurons. Regular exercise contributed to successful memory consolidation while memory persistence failed following repeated treatment with the AMPK inhibitor. Repeated treatment with the AMPK inhibitor also caused a reduction in AMPK activity in hippocampal CA1. Notably, the performance of mice which underwent treadmill running was attenuated by the AMPK inhibitor during the probe test on day 28 (Figure 3Ab), thus suggesting the important role of AMPK in exercise-induced memory retention.

Regular exercise enlarged the dendrites of neurons in the dorsal hippocampal CA1 area, suggesting that regular exercise is likely to affect structural plasticity in the temporoammonic-CA1 pathway and Schaffer collateral (SC) synapses in the dorsal hippocampal CA1 region. Interestingly, AMPK inactivation resulted in the retraction of neuronal dendrites. Accordingly, these results support our hypothesis that exercise-induced AMPK activation enlarges or restores dendrites of hippocampal CA1 neurons during chronic stress, thereby improving memory consolidation. Collectively, the mild activation of AMPK through regular exercise can prevent chronic stress-induced impairments in memory persistence and dendritic shrinkage among hippocampal CA1 neurons.
